# The effect of intergenerational rearing on young children’s self-control: The intervention effect of sports games

**DOI:** 10.3389/fpsyg.2022.1051341

**Published:** 2022-12-02

**Authors:** Boqian Sun, Haiying Quan, Ye Zhang

**Affiliations:** Institute of Physical Education, Liaoning Normal University, Liaoning, China

**Keywords:** intergenerational rearing, self-control, delayed gratification, sports games, intervention study

## Abstract

**Objective:**

To investigate the effect of different rearing arrangements on children’s self-control and verify the promoting effect sports games might have on children’s self-control in intergenerational rearing.

**Methods:**

A total of 72 intergenerational rearing children and 66 non-intergenerational rearing children were included in Experiment 1, in which the delay-of-gratification task was used to examine the differences in self-control among children with different rearing arrangements. In experiment 2, 70 intergenerational rearing children were included as subjects, and sports games were used to improve their self-control.

**Results:**

The results of experiment 1 showed that compared with non-intergenerational rearing children, the self-control ability of intergenerational rearing children was poor. In experiment 2, it was found that after the intervention with sports games, the self-control ability of the children in the intergenerational rearing group was effectively improved.

**Conclusion:**

Although intergenerational rearing arrangements have a certain negative impact on the self-control ability of children, the sports game intervention can be used to effectively develop the self-control ability of intergenerational rearing children.

## Introduction

Self-control refers to an individual’s ability to restrain, regulate, and alter impulses, desires, and habitual responses (Baumeister and Heatherton, 1996). The critical period for individuals to develop self-control occurs sometime at the age of 4 (Yang and Shen, 2013), and some studies have even shown that children’s self-control at this time can predict their future cognitive and mental health, academic achievement, income in adulthood, and job satisfaction (Mischel et al., 2011; Duckworth et al., 2013; Converse et al., 2014). Toddlers with low self-control, who not only show less persistence and focus at a young age, but are more hyperactive and impulsive, were found to have a greater tendency to be in poorer health, with lower financial stability, and more prone to crime 30 years later (Moffitt et al., 2011).

Studies have shown that children’s self-control is affected by factors such as the growth environment and their physical fitness. First, Lawler et al. (2019) showed that compared with young children from ordinary families, internationally adopted children had delayed self-control development, which indicates that young children’s self-control is profoundly affected by the growth environment. As the key factors in children’s growth environment, the family structure and rearing arrangements may also restrict the development of children’s self-control. In particular, Chinese families generally have a culture of intergenerational rearing (Chen et al., 2022). In this kind of family structure and rearing arrangement, many elderly grandparents bear the responsibility of raising their grandchildren due to traditional customs or out of practical problems such as their children working in different places. However, this rearing arrangement often negatively affects the development of children’s theory of mind, academic performance, health behavior, emotional stability, etc. (Hayslip and Kaminski, 2005; Edwards and Daire, 2006; Bramlett and Blumberg, 2007; Guo, 2014). Moreover, self-control is also positively correlated with individual physical fitness (Boat and Cooper, 2019). Compared with non-intergenerational rearing children, intergenerational rearing children not only lag behind in the development of gross motor and fine motor (Zhang et al., 2015) but also lag behind in the overall health level (Man et al., 2019). Therefore, low physical quality is also likely to lead to poor self-control in intergenerational rearing children. Accordingly, we hypothesized that intergenerational rearing children would have poorer self-control compared to children not exposed to intergenerational rearing (Hypothesis 1).

From the point of view of plasticity in relation to self-control ability, reasonable and effective intervention in the critical period of children’s self-control development can positively affect the development of individual self-control. For example, Sun et al. (2015) used games to train children’s self-control. After training the average amplitudes of the N2 and P3 components of children in the NoGo task were significantly reduced. Under NoGo conditions, these two ERP components are thought to be related to inhibitory control ability (Folstein and Van Petten, 2008; Smith and Douglas, 2011), and the smaller the amplitude, the better the inhibitory control ability of the individual. Therefore, the results of this study indicated that game training could promote the development of brain systems associated with children’s self-control. In their study, Lawler et al. (2019) also found that children’s delayed gratification, inhibitory control, and selective attention were improved through mindfulness training and play training based on developmental executive control.

Previous studies have shown that physical activity can also positively promote the development of self-control. For example, Huang et al. (2020) reported that 30 min of moderate-intensity aerobic exercise could significantly increase the activation of l-DLPFC (left dorsolateral prefrontal cortex), an important part of the executive control network, which is closely related to the individual’s self-control ability, in obese subjects. The increased activation in this region indicates improved self-control ability of an individual. Another study by Chaddock-Heyman et al. (2013) found that 60 min of physical activity five times a week could effectively reduce the activation level of the right prefrontal area, which is closely related to cognitive control in children aged 8–9 years old, thus indicating that physical activity promotes the development of cognitive control in children. However, compared with children without intergenerational rearing background, the level of sports participation of children with intergenerational care is significantly lower (Zhang et al., 2017). Therefore, in order to make sports activities more interesting, it is necessary to increase the zeal to participate among children with intergenerational care. As previous studies have suggested that sports games as an intervention method have a positive effect on children’s physical and mental development (Quan and Ma, 2014; Zhao and Zhang, 2018; Yu et al., 2020), we hypothesized that sports games could improve the self-control ability of intergenerational rearing children (Hypothesis 2).

## Experiment 1: Differences in children’s self-control abilities with different rearing arrangements

### Research design

The present study adopted a single-factor between-subject design. The independent variable was the rearing arrangement, which included two levels of intergenerational rearing and non-intergenerational rearing. The dependent variable was the self-control ability of the child. In terms of the selection of evaluation indicators and measurement methods for children’s self-control, this study was mainly based on the concept of delay-of-gratification proposed by Mischel et al. They argued that delay-of-gratification is the core component and important skill for children’s self-control, essential for children’s socialization, as well as an important component of emotional regulation, and a positive personality force that accompanies a person’s life (Mischel et al., 1988). Therefore, we used the performance of young children in the delay-of-gratification task as an index to measure their self-control ability and compared the influence of different rearing arrangements on individual self-control ability according to children’s performance in the delay-of-gratification task.

### Subjects

A total of 72 intergenerational rearing children were recruited from two urban kindergartens; 66 non-intergenerational rearing children were also enrolled as a control group. Table 1 shows the details of all the children; there was no significant difference in age and sex composition between the two groups. In this study, intergenerational rearing means that the maternal or paternal grandparents take the primary responsibility for raising children; non-intergenerational rearing means that parents assume the primary responsibility for raising children. Data on child-rearing arrangements and whether parents agreed to participate in the follow-up study were collected *via* a telephone survey conducted by kindergarten teachers who interviewed parents of young children (the teacher was assigned by the kindergarten and did not participate in the data collection process of the subsequent experimental study). G-power 3.1 was used for the calculation of the sample size in this study. The power of the test was taken as 1 − *β* = 0.80, while the one-tailed test *α* = 0.05 and the effect size *d* = 0.65. A total of 60 subjects were required for the independent-samples *t*-test. Parents signed a written informed consent form, including a detailed description of the experimental procedure and the possible risks.

**Table 1:** The distribution and the average age of the children according to different rearing arrangements.

Rearing arrangements	Number	Gender		Average age (months) (*M ± SD*)
		Male	Female	
Intergenerational support	72	39	33	50.28 ± 1.60
Non-intergenerational support	66	36	30	50.45 ± 1.85
Total	138	75	63	50.36 ± 1.73

The Ethics committee of Liaoning Normal University approved the experimental protocol, which was in accordance with the ethical standards of the 2013 Declaration of Helsinki.

### Research materials

The study was carried out in two laboratories separated by one-way mirrors. The room where the children were accommodated had a mirror surface, a square table for children, two small chairs for children and a chair for adults, a doorbell, two Kinder eggs, a stopwatch, and a video recorder.

### Research procedures

After the parents brought the children to the laboratory, they were first asked to sign the informed consent form related to the research. Afterward, they read a picture book story together. The experiment officially began when the examiners made sure that the children got familiar with the environment and felt comfortable enough. In the delayed gratification task, the examiner first introduced the function of the doorbell to the child, telling him that when he rang the doorbell, the examiner outside the room would immediately return to the room. To ensure that the children fully understood the function of the doorbell, after listening to the main test explanation, they were asked to try it three times. After that, the examiner showed two Kinder eggs to the children and told them that he would put one on the table and take the other with him when he left the room. If the child could wait until the main examiner returned, the child would get both of these eggs, but if he decided to press the doorbell to let the main examiner return in advance, he would only get one Kinder egg. In order to ensure that the children fully understood the experimental requirements, after the main test was finished, the children were asked three questions: ① “If you wait until I come back, how many Kinder eggs can you get?” ② “If you want me to come back sooner, what do you need to do?” ③ “If you ring the doorbell and let me come back early, how many Kinder eggs can you get?” If the children could not answer the above questions correctly, the examiner further explained the experimental requirements to them. When the children could answer the above questions correctly, their parents and examiner would leave the room and observe the children’s behavior in the adjacent room through a single-sided mirror. The maximum departure time was 15 min.

### Timing and coding

Delay time recording: the timing started at the moment when the main examiner left the room. When one of the following three situations occurred, the timing was stopped: ① after 15 min, the main examiner voluntarily returned to the room; ② the child rang the doorbell, after which the main examiner returned to the room and gave the child an instant reward; ③ the child picked up or ate the Kinder eggs in the middle of the experiment, thus violating the rules.

Delay strategy coding: according to the relevant coding strategy reported in Yang’s research (Yang et al., 2005), we adopted the time sampling observation method, where every 15 s, the delay behavior of the children captured by the camera was coded accordingly, i.e., when the children showed any coding behavior, they scored 1 point under the corresponding strategy. The coding strategies were as follows: ① attempting to ring the bell: the child tried to ring the bell but did not ring it; ② negative behavior: the child manifested negative behaviors such as tantrums, crying, etc.; ③ seeking parents: the child shouted and looked for the parent; ④ goal-seeking: the children manifested behaviors that were actively focused toward the reward, such as approaching the Kinder Eggs, but not picking them up or eating them; ⑤ avoiding the doorbell: the children pushed the doorbell away; moved around on the chair, played with their own hands, etc.; ⑥ leaving the seat: the child left the chair and moved around the room; ⑦ sitting quietly: the child sat quietly on the chair; ⑧ Task talk: the child talked to himself about the reward or having to wait for rewards, e.g., “When will that lady come back?” ⑨ Non-task self-talk: the children talked about topics not related to rewards or having to wait for rewards, e.g., the children retold the previous picture book stories; ➉ self-reinforcement: the children encouraged themselves to be patient, e.g., “I must hold on!” To ensure the reliability of the encoding, all video encodings were completed by two researchers. Before the formal coding, the raters were trained on how to code, and the video was coded when the two raters reached 95% consistency. When there was a discrepancy between the two raters, the final encoding was determined by the two raters through discussion.

### Data collection and statistical processing

The experimental data mainly came from the analysis of the video. SPSS19.0 was used to conduct the independent sample *t*-test for the data in line with the normal distribution and the Mann–Whitney *U* test in conformity with the normal distribution.

### Results

#### The influence of rearing arrangements on the delay time of young children

In Experiment 1, the independent sample *t*-test was carried out with the rearing mode (two levels of intergenerational rearing and non-intergenerational rearing) as the independent variable and the child delay time as the dependent variable (Table 2). The delay time in the delayed gratification task was significantly longer for non-intergenerational rearing children than for intergenerational rearing children (*t* (136) = 2.179, *p* < 0.05, Cohen’s *d* = 0.371).

**Table 2:** The *t*-test results of the delay time of young children under different rearing arrangements (unit: s).

Rearing arrangements	*M*	*SD*	*t*	*df*	Value of *p*	95% confidence interval
Intergenerational support (*n* = 72)	453.722	200.447	2.179	1	0.031	6.957–143.871
Non-intergenerational support (*n* = 66)	529.136	206.030				

#### The influence of rearing arrangements on children’s delay strategies

In order to better understand the development level of children’s delay strategies, Yang et al. (2005) divided the delay strategies in children’s delay-of-gratification task at different levels according to Mischel’s theory of cold and heat systems, where “cold system” mainly includes various distraction activities, as these can effectively divert the attention of young children to the arousal characteristics of rewards, thus furthering the delay. Therefore, these are considered to be more advanced strategies. On the contrary, the “thermal system,” which mainly shows that young children keep their attention fixed on the reward, is considered a lower-level strategy because of its focus on arousal characteristics and thus is not conducive to delay (Metcalfe and Mischel, 1999). According to the above principles, the 11 coded delay strategies were divided into four levels from low to high, where level 1 was meaningless strategies, including attempts to ring the bell and negative behavior strategies; level 2 was seeking strategies, including parent-seeking and goal-seeking strategies; level III were self-distraction and problem-solving strategies, including doorbell avoidance, action distraction, sedentary and sedentary strategies; level IV was self-verbal control strategies, including task speech, non-task speech and self-reinforcing strategies (Yang et al., 2005).

The independent sample *t*-test was carried out with the rearing arrangements (two levels of intergenerational care and non-intergenerational care) as the independent variable and the scores of the children’s delay strategy levels as the dependent variable. The results showed (Table 3) no difference in the use of level I meaningless strategies between children raised by intergenerational and non-intergenerational rearing (*t* (136) = 1.018, *p* > 0.05, Cohen’s *d* = 0.173); in the use of level II seeking strategies, there was also no difference between children in relation to rearing arrangements (*t* (136) = 0.743, *p* > 0.05, Cohen’s *d* = 0.127); with reference to the use of self-distraction and problem-solving strategies at level III, children who were reared between generations were significantly less prone to use these strategies than children who were not reared between generations (*t* (136) = 3.656, *p* < 0.01, Cohen’s *d* = 0.621). Since the original data of Level IV for non-generational rearing and generational rearing children did not coincide with normal distribution, the Mann–Whitney *U* test was used to test the difference between the two groups of data. The results showed there was no difference between the two rearing arrangements in the use of self-verbal control strategies at Level IV (Mann–Whitney *U* = 0.080, *p* > 0.05, Cohen’s *d* = 3.402). Based on the above results, it can be seen that in the delayed gratification task, the delay of time and high-level delay strategies were less practiced by children with generational rearing than in non-generational rearing. Their ability to control was worse than children with non-generational rearing, which supported research hypothesis 1. Accordingly, improving the self-control ability of these intergenerational rearing children is of great importance, so we discussed this issue in Experiment 2, where we proposed a sports game activity plan that could effectively promote the self-control abilities in intergenerational rearing children.

**Table 3:** The *t*-test results of the scores of each level of children’s delay strategy under different rearing arrangements.

Delay strategy	Rearing arrangements	*M*	*SD*	*t*	*df*	Value of *p*	95% confidence interval
Level I nonsense strategy	Generation-skipping (*n* = 72)	2.486	1.343	1.018	1	0.311	−0.228-0.710
	Non-generational support (*n* = 66)	2.727	1.442				
Level II seeking strategies	Generation-skipping (*n* = 72)	18.569	8.099	0.743	1	0.459	−1.699-3.741
	Non-generational support (*n* = 66)	19.591	8.040				
Level III self-distraction, problem solving strategies	Generation-skipping (*n* = 72)	7.597	5.056	3.656	1	0.000	1.555–5.220
	Non-generational support (*n* = 66)	10.985	5.824				

## Experiment 2: The effect of sports games on the self-control ability of intergenerational rearing children

### Research design

The study adopted a 2 (group) × 2 (test type) mixed design, in which the group was the independent variable between groups, including two levels of the experimental group and the control group, and the test type was the independent variable within the group, including the pre-test and the post-test level. The dependent variable was the child’s ability to self-control. The purpose of Experiment 2 was to explore whether sports games can promote the self-control ability of intergenerational rearing children.

### Subjects

A total of 70 intergenerational rearing children were selected from two urban kindergartens, and the children from one kindergarten were randomly assigned to the experimental group and the children from the other kindergarten to the control group. The children’s details are shown in Table 4; there was no significant difference in age and sex composition between the two groups. G-power 3.1 was used for the calculation of the sample size in this study. The power of the test was taken as 1 − *β* = 0.80, while *α* = 0.05 and partial *η*^2^ = 0.06.64 subjects were required for the repeated measures analysis.

**Table 4:** Distribution and average age of children in different groups.

Group	Number	Gender		Average age (months) (*M ± SD*)
		Male	Female	
Test group	33	17	16	50.546 ± 1.697
Control group	37	22	15	50.027 ± 1.554
Total	70	39	31	50.271 ± 1.632

### Research materials

#### Dependent variable measurement

The measures of children’s self-control ability were the same as those used in Experiment 1.

#### Intervention

##### Intervention time and place

The intervention period lasted 4 weeks in total, from 9:30 a.m. to 10:30 a.m. from Monday to Friday in the playground outside the kindergarten. The activities of the children in the experimental group included sports games, while the children in the control group performed basic gymnastics activities for children in the same period of time, i.e., the children in the control group performed 5 basic gymnastics activities for 1 h each time every week (15 min rest was included during the activities of the two groups of children).

##### Intervention organization

The physical activity of the experimental group and the control group was conducted by kindergarten teachers. Before the experiment, the researcher trained the teachers in the experimental group, clarifying the matters needing attention so that the teachers would become familiar with the operation process of sports game activities. During the game, the teachers led the children in the experimental group into the pre-arranged game place and carried out activities according to the predetermined sports game activity plan. Children in the control group completed basic gymnastics under the guidance of teachers.

##### Intervention materials

According to the mechanism and classification of self-control training (Zhang and Zhang, 2017), in order to ensure that the implemented sports games could effectively promote the self-control ability of intergenerational rearing children in the sports game intervention stage, it was necessary to establish some rules in sports games setting content such as inhibiting individual dominant responses, changing original habits, and using self-control resources. On the other hand, we designed physical activity content based on the motor development stage of 4-year-old children and the theory of zone of proximal development to ensure that children can not only complete the designed task, but that it can also promote their further development. The study designed a total of 10 sports games, such as “Anti-Password” and “123 Wooden Man.” The following is a detailed introduction to the sports game design ideas and this study’s specific intervention implementation process, taking “Send Egg Baby Home” as an example.

Equipment: some silicone spoons, some table tennis balls, four plastic buckets, and one hen toy.

Classroom routine: Form the team and count the number of people.

Task: quickly pass the obstacles set on the ground, and try to ensure that the table tennis balls in the silicone spoons, which you are not supposed to touch with your hands, do not fall.

Game rules: Children are divided into two equal groups (if the number of children participating in the activity is odd, the teacher can also participate in the game), and asked to stand in two rows behind the starting point (to raise the children’s awareness of obeying the rules). Each child holds a silicone spoon with a table tennis ball, and after the two teams of children hear the start signal, they quickly pass the obstacles set up in turn and try their best to keep the table tennis ball from falling until they put the ball into the small bucket at the end of the room. The group with the shorter time to complete the task wins, and the group that loses does five squats.

##### Exercise intensity

The intensity of the game in the study is guaranteed to be of moderate exercise intensity, i.e., the heart rate should reach (220-age) × (60%–69%). During the activities, children wear the Polar heart rate belt, which monitors the heart rate; the average heart rate is controlled at 125–140 beats/min (Pan et al., 2016).

### Procedures

Pre-test: delay-of-gratification task → sports game intervention stage → post-test: delay-of-gratification task (all the same as in Experiment 1).

In the above process, children were required to complete the self-delayed gratification task before and after the physical activity intervention to assess their self-control ability. The procedure and requirements of the delay-of-gratification task were the same as the task in experiment 1, and they mainly included four steps: first, parents were required to sign a consent form, and then the experimenter read a story together with young children, after which he introduced children to the doorbell function and activities of requirements. Finally, the formal testing began after ensuring that children understood the task requirements completely.

### Data collection and statistical analysis

The experimental data were mainly derived from the analysis of the video. The related repeated measures analysis of variance was carried out by SPSS 19.0.

### Results

#### The effect of sports games on the delay time of young children

**Figure 1:**
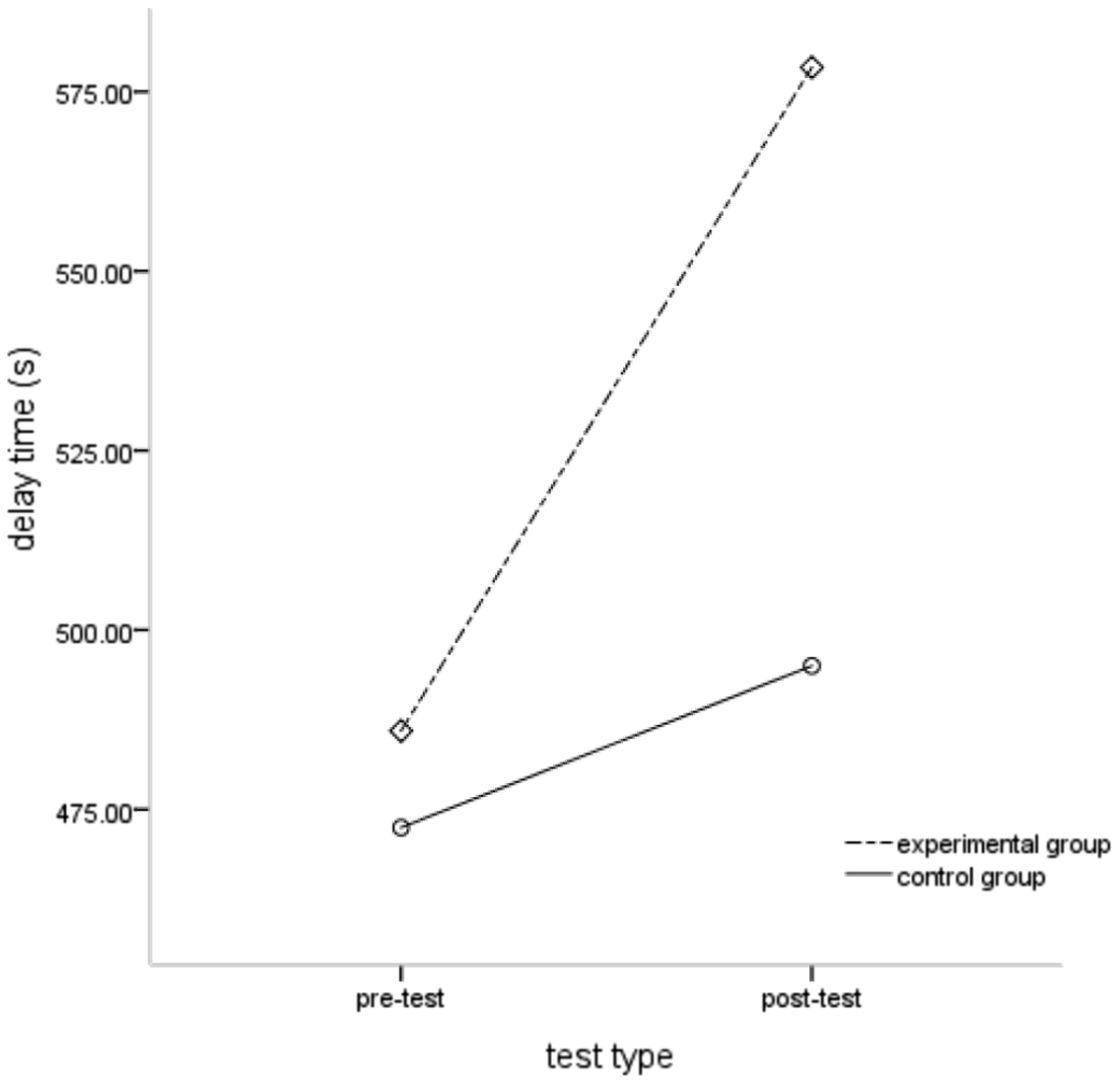
Comparison of delay time between experimental group and control group.

Taking the delay time of young children as the dependent variable, 2 (group) × 2 (test type) repeated measures analysis of variance was performed, and the results showed that the main effect of the group was not significant, *F*(1, 68) = 1.427, *p* > 0.05, partial *η*^2^ = 0.021; the main effect of test type was significant, *F*(1, 68) = 51.587, *p* < 0.001, partial *η*^2^ = 0.431; the interaction between the group and test type was significant, *F*(1, 68) = 19.068, *p* < 0.001, partial *η*^2^ = 0.219. Further simple effect analysis showed (Figure 1) that there was no significant difference in the pre-test delay time between the experimental and control groups. The time was (472.460 ± 197.972) s, *F*(1, 68) = 0.095, *p* > 0.05, partial *η*^2^ = 0.001; but in the post-test, the delay time of the children in the experimental group was significantly longer than that of the children in the control group. The post-test delay time of the experimental group was (578.394 ± 144.268) s, and the pre-test delay time of the control group was (495.000 ± 175.293) s, *F*(1, 68) = 4.655, *p* < 0.05, partial *η*^2^ = 0.064.

#### The influence of sports games on the delay strategies of young children

Table 5 shows the descriptive statistical results of delay strategy scores at different levels in children from different groups.

**Table 5:** Descriptive statistical results of delay strategy scores at different levels in children from different groups.

Delay strategy	Group	Pre-test		Post-test	
		*M*	*SD*	*M*	*SD*
Level I nonsense strategy	Experimental group (*n* = 33)	2.485	1.302	2.273	1.069
	Control group (*n* = 37)	2.757	0.983	2.811	0.967
Level II seeking strategies	Experimental group (*n* = 33)	19.455	7.080	19.091	5.897
	Control group (*n* = 37)	18.027	8.050	18.676	7.461
Level III self-distraction, problem solving strategies	Experimental group (*n* = 33)	8.394	3.848	15.061	4.854
	Control group (*n* = 37)	9.054	4.784	9.676	4.391
Level IV self-verbal control strategies	Experimental group (*n* = 33)	1.576	1.146	1.636	0.895
	Control group (*n* = 37)	1.162	1.143	1.432	0.801

Taking the level I meaningless strategy of children’s delayed gratification as the dependent variable, a 2 (group) × 2 (test type) repeated measures analysis of variance was performed. The results showed that the main effect of the group was not significant, *F*(1, 68) = 2.891, *p* > 0.05, partial *η*^2^ = 0.041; the main effect of test type was not significant, *F*(1, 68) = 0.600, *p* > 0.05, partial *η*^2^ = 0.009; the interaction between the group and test type was not significant, *F*(1, 68) = 1.701, *p* > 0.05, partial *η*^2^ = 0.024 (Table 6).

**Table 6:** Analysis of variance results of different levels of children’s delay strategy scores in different groups.

Delay strategy	Variable	Type III sum of squares	*df*	Mean square	*F*	Value of *p*
Level I nonsense strategy	group	5.722	1	5.722	2.891	0.094
	test type	0.218	1	0.218	0.600	0.441
	Group × Test Type	0.618	1	0.618	1.701	0.197
Level II seeking strategies	group	29.616	1	29.616	0.297	0.588
	test type	0.708	1	0.708	0.176	0.676
	Group × Test Type	8.937	1	8.937	2.218	0.141
Level III self-distraction, problem solving strategies	group	194.696	1	194.696	5.538	0.022
	test type	463.275	1	463.275	88.735	0.000
	Group × Test Type	318.703	1	318.703	61.044	0.000
Level IV self-verbal control strategies	group	3.326	1	3.326	2.090	0.153
	test type	0.955	1	0.955	2.194	0.143
	Group × Test Type	0.383	1	0.383	0.881	0.351

Taking the children’s delayed gratification level II seeking strategy as the dependent variable, a 2 (group) × 2 (test type) repeated measures analysis of variance was performed. The results showed that the main effect of the group was not significant, *F*(1, 68) = 0.297, *p* > 0.05, partial *η*^2^ = 0.032; the main effect of test type was not significant, *F*(1, 68) = 0.176, *p* > 0.05, partial *η*^2^ = 0.003; the interaction between the group and test type was not significant, *F*(1, 68) = 2.218, *p* > 0.05, partial *η*^2^ = 0.032 (Table 6).

**Figure 2:**
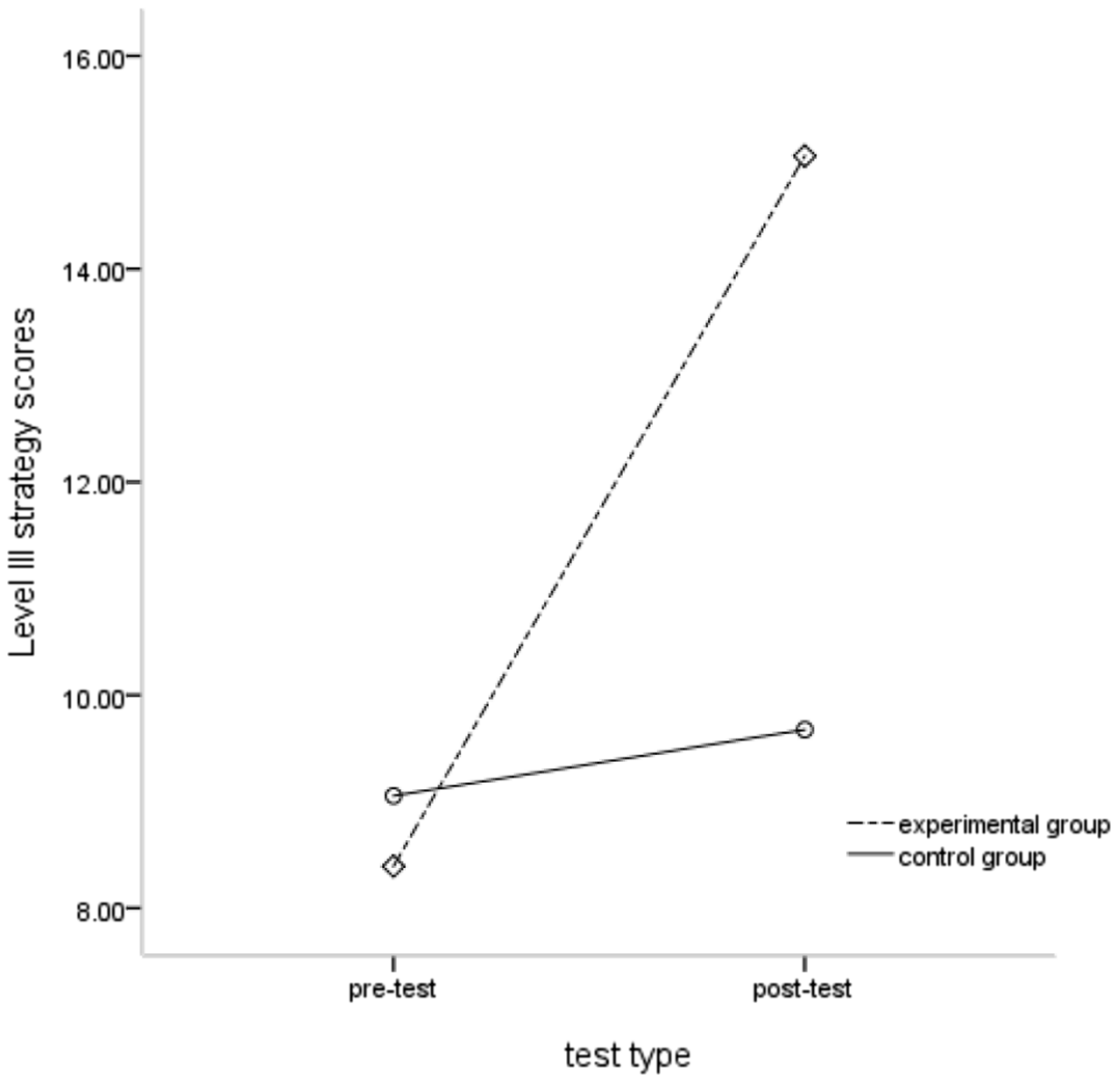
Comparison of Level III strategy scores between the experimental group and the control group.

Taking the level III self-distraction and problem-solving strategies of children’s delayed gratification as dependent variables, a 2 (group) × 2 (test type) repeated measures analysis of variance was performed. The results showed that the main effect of the group was significant, *F*(1, 68) = 5.538, *p* < 0.05, partial *η*^2^ = 0.075; the main effect of test type was significant, *F*(1, 68) = 88.735, *p* < 0.001, partial *η*^2^ = 0.566; the interaction between the group and test type was significant, *F*(1, 68) = 61.044, *p* < 0.001, partial *η*^2^ = 0.473. Further simple effect analysis showed (Figure 2) that in the pre-test, there was no difference in the use of the Level III strategy between the experimental group and the control group, *F*(1, 68) = 0.398, *p* > 0.05, partial *η*^2^=0.006; but in the post-test, the use of level III strategy in the experimental group was significantly higher than that in the control group, *F*(1, 68) = 23.753, *p* < 0.001, partial *η*^2^ = 0.259 (Table 6).

Taking the level IV self-verbal control strategy of children’s delayed gratification as the dependent variable, a 2 (group) × 2 (test type) repeated measures analysis of variance was performed, revealing no significance in the main effect of the group *F*(1, 68) = 2.090, *p* > 0.05, partial *η*^2^ = 0.013; the main effect of test type was not significant, *F*(1, 68) = 2.194, *p* > 0.05, partial *η*^2^ = 0.031; the interaction between the group and test type was not significant, *F*(1, 68) = 0.881, *p* > 0.05, partial *η*^2^ = 0.013 (Table 6).

Based on the above results, it can be seen that after the sports games, the children in the experimental group performed better than the children in the control group in terms of delay time and high-level delay strategies in the delayed gratification task. The self-control of intergenerational rearing children showed positive changes, thus providing support for our research hypothesis 2.

## Discussion

### The influence of rearing arrangements on children’s self-control

Results in the present study showed that compared with non-generational rearing children, intergenerational rearing children used less delay time and high-level delay strategies in the delayed gratification task, thus indicating that intergenerational rearing children had poorer self-control ability than children who were reared by different generations, which is not consistent with the results of previous studies. In his study, Guo (2014) found that, unlike adults’ subjective evaluation of children’s self-control ability, by measuring the inhibitory function of children with different rearing arrangements, no difference was found. There was a difference in the self-control ability of children exposed to intergenerational rearing and those exposed to non-generational rearing. At the same time, the study also pointed out that potential differences in the self-control ability of children raised between generations and children raised in non-generations were not due to the poor development of executive function in the former but because of the permissive parenting strategies used by grandparents that failed to motivate children to perform beyond the level of self-control they already possess. However, from the results of this study, in the delayed gratification task, intergenerational rearing children had a shorter delay time and used less high-level delay strategies, which indicated that intergenerational rearing children performed worse in delayed gratification tasks. This is likely due to their poor self-control development, preventing them from using higher-level delay strategies. The poor development of self-control in intergenerational rearing children is likely due to the lack of self-support. Numerous studies have shown that in the process of educating young children if the caregivers can respect and support the autonomy needs of young children, this will promote the development of children’s self-control. Yet, compared with parents, grandparents tend to pay less attention to children’s psychological and emotional needs, and tend to regard children’s opposing views as disrespectful (Hayslip and Kaminski, 2005), which leads to the lack of autonomous support for children in intergenerational rearing, and hinders their development of self-control.

This study further supports the Hot/Cool System Framework constructed by Metcalfe and Mischel (1999). In this theoretical framework, the rapid emotional processing and the response to end waiting when reward stimuli awaken children are called the “thermal system.” The self-controlled response based on the abstract features of reward stimuli is called “cold system.” The former develops earlier than the latter but weakens the individual’s ability to control impulses, while the latter enhances the individual’s ability to resist temptation. In this study, due to the high level of delayed strategies used by non-intergenerational rearing children, we were often able to shift their attention from the reward, convert the hot representation into the cold representation, and improve the control ability of the children to resist the temptation of the reward, thus increasing the delay time. However, due to the low level of delay strategies used by young children, they cannot be distracted from the reward and cannot realize the transformation of the hot system to the cold system, thus weakening the young children’s ability to control impulses and shortening the delay time.

Overall, the impact of intergenerational rearing on children’s self-control is likely to be direct and fundamental, rather than simply affecting their explicit behavior. Still, this speculation needs to be further verified in subsequent studies. Therefore, future studies on the self-control ability of young intergenerational rearing children should focus on the brain mechanisms so as to better explain how the rearing arrangements affect the self-control ability in young children.

### The effect of sports games on the self-control ability of intergenerational rearing children

Play is a power tool for children, during which they obtain opportunities for new cognitive structure development and emotional development, as well as achieve assimilation and meet their own needs (Piaget and Inhalder, 1980). Sports games are not only similar to general games, but they can also positively affect the development of children’s physical and mental health. The sports game intervention program designed in this study increases the delay time and the use of high-level delay strategies in the self-delayed gratification task of intergenerational rearing children, which is the result of the effect of sports game intervention on children’s self-control.

First of all, in the design process of sports games, the research is based on physical activities, based on the actual situation of children’s own development, and according to the mechanism and classification of self-control training set in sports games (Zhang and Zhang, 2017). Sports games focus on suppressing individual dominant responses, changing original habits, and using self-control resources. For example, in sports games, young children need to break their original habit of using sharp-handed spoons, and use non-handed spoons to complete related tasks in the game, which makes children have to constantly use their own control resources. Just as muscle strength can be improved during repeated strength training, self-control resources can also be developed during repeated mobilization, which is consistent with research related to self-control training (Muraven and Baumeister, 2000).

Second, in sports games, children are provided with more opportunities for social interaction and conditions for the use of strategic skills. When children participate in basic gymnastics, this relatively simple aerobic exercise does not require the use of advanced cognitive functions, nor does it require the body to complete complex coordinated movements, so children’s self-control ability cannot be exercised. In sports games, children need to cooperate with each other, adopt behavioral strategies, and coordinate activities of the whole body (Pesce et al., 2009), which requires more self-control processes. Therefore, compared with simple sports, sports games are more likely to promote self-development of control ability (Zhu, 2015).

Finally, sports games usually include rigid rules. Under such rules, if they want to complete game tasks, they must suppress interference and temptation and insist on the behavior needed to complete the task. During this process, their self-control ability also improves. For example, in the game “Red Light, Green Light, Little White Light,” the child can only move forward when the person is facing the wall, and when the person stops talking and turns to face the child, the child must continue standing still. On the one hand, the child wants to quickly reach the endpoint; on the other hand, they must control their own behavior so as to obey the rules. In this situation, children further develop their self-control ability.

### Research limitations and prospects

In order to reduce or avoid the appearance of the Hawthorne effect, during the intervention experiment, children in two different kindergartens were randomly assigned to the experimental group and the control group and were given the same physical activity as described above for the experimental group and control group. Although this method can reduce or avoid the Hawthorne effect, there may be differences in the environment, conditions, and teachers of the two kindergartens, which may affect young children. Therefore, in future research, not only should we consider avoiding the appearance of the Hawthorne effect, but we should also further consider eliminating other possible interference variables.

## Conclusion

The results showed that although the self-control ability in children with the intergenerational care was poorer than in those with non-intergenerational care, the intervention of sports games could not only prolong the delay time of the self-delayed gratification task but could also effectively increase the frequency of use of high-level delay strategies tasks, thus indicating that sports games have a significant effect on the development of self-control in intergenerational rearing children, and provide effective practical method support for the development of self-control in intergenerational rearing children.

## Data availability statement

The original contributions presented in the study are included in the article/Supplementary material, further inquiries can be directed to the corresponding author.

## Ethics statement

The studies involving human participants were reviewed and approved by Liaoning Normal University Ethics Committee. Written informed consent to participate in this study was provided by the participants’ legal guardian/next of kin.

## Author contributions

BS: conceptualization, methodology, formal analysis, data curation, and writing–original draft. HQ: methodology, writing–review and editing, and supervision. YZ: data analysis, writing -review and editing. All authors contributed to the article and approved the submitted version.

## Funding

This work was supported by High-End Scientific Research Achievements Cultivation Funding Program of Liaoning Normal University in 2021 (21GDW003).

## Conflict of interest

The authors declare that the research was conducted in the absence of any commercial or financial relationships that could be construed as a potential conflict of interest.

## Publisher’s note

All claims expressed in this article are solely those of the authors and do not necessarily represent those of their affiliated organizations, or those of the publisher, the editors and the reviewers. Any product that may be evaluated in this article, or claim that may be made by its manufacturer, is not guaranteed or endorsed by the publisher.
